# Zoonotic disease knowledge, attitudes, and livestock biosecurity practices among community members in Ada East District, Ghana: a concurrent mixed-methods study

**DOI:** 10.1186/s42522-026-00221-8

**Published:** 2026-06-16

**Authors:** Kirstin P. West, Marta A. Kisiel, Kristin K. Sznajder, Hannah E. Sauve, Leonard Baatiema, Godwin Dogbey, Abebayehu N. Yilma

**Affiliations:** 1 Department of Public Health Sciences, Pennsylvania State University College of Medicine, Hershey, PA USA; 2 Institute of Energy and the Environment, Pennsylvania State University, University Park, PA USA; 3 Department of Global Health, School of Public Health and School of Medicine, University of Washington, Seattle, WA USA; 4 Department of Medical Sciences, Occupational and Environmental Medicine, Uppsala University, Uppsala, Sweden; 5 Department of Epidemiology, School of Public Health, Boston University, Boston, MA USA; 6 Department of Health Policy Planning and Management, College of Health Sciences, School of Public Health, University of Ghana, Legon, Ghana; 7 School of Veterinary Sciences, University for Development Studies, Nyankpala, Ghana

**Keywords:** Zoonoses, Knowledge, attitudes and practices, Biosecurity, Livestock, One health, Ghana

## Abstract

Zoonotic diseases disproportionately burden settings where humans and livestock coexist, yet community-level knowledge, attitudes, and practices (KAP) remain poorly characterized in West Africa. This study aimed to assess KAP regarding zoonotic diseases and document household biosecurity gaps among livestock-keeping community members in Ada East Distrct in Ghana. A concurrent mixed-methods design was employed, integrating a quantitative cross-sectional survey with a qualitative focus group discussion. Livestock-keeping adults were recruited from five health facilities in Ada East District. A structured survey measured knowledge, attitudes, and livestock-handling practices. Composite KAP scores were dichotomized using a modified Bloom’s cutoff (≥60%). Multivariable logistic regression analyses were conducted to identify factors associated with good knowledge, positive attitudes, and good practices. A focus group discussion (FGD) explored perceptions of transmission, livestock management, and outbreak preparedness. Among 252 survey participants, 66.3% demonstrated good overall knowledge of zoonotic diseases; however, only 10.6% had good knowledge of Ebola virus disease symptoms. Positive attitudes were observed in 44.0% of respondents, while 86.9% reported good livestock-handling practices. Education beyond primary school was independently associated with higher odds of good knowledge (adjusted odds ratio (aOR) 1.97, 95% confidence intervals (CI) 1.05–3.69). Animal-related occupations were associated with lower odds of positive attitudes (aOR 0.46, 95% CI 0.25–0.85) but higher odds of good practices (aOR 3.58, 95% CI 1.26–11.8). FGD (*n* = 8) identified bats and sick animals as transmission sources, described economic barriers, and expressed variable beliefs regarding prevention and stigma. These findings highlight the need for locally tailored, One Health–oriented risk communication and biosecurity interventions targeting groups with lower educational attainment and limited access to animal health services.

**Supplementary information:**

The online version contains supplementary material available at 10.1186/s42522-026-00221-8.

## Introduction

Zoonotic diseases are infections transmitted between animals and humans and account for a substantial share of emerging and re-emerging infectious disease events globally [1–4]. Recurrent zoonotic outbreaks in the twenty-first century, including pandemic influenza and coronaviruses, have highlighted how land-use change, agricultural expansion, intensification of animal production, and increased human–animal contact elevate spillover risk [5–8]. Once spillover occurs, global connectivity through the movement of people, animals, and animal products accelerates disease transmission across regions and borders [6, 9]. Because these risks originate at the human-animal-environment interface, effective prevention and response increasingly require One Health approaches that coordinate human, animal, and environmental sectors for surveillance, prevention, and control [10, 11].

Sub-Saharan Africa is frequently highlighted in global analyses as a region where ecological change, reliance on animal-based livelihoods, and expanding production systems intersect to create conditions conducive to zoonotic emergence. However, mapped “hotspots” are also influenced by surveillance and reporting capacity [2, 12, 13]. Close proximity between humans and animals, limited veterinary services, and informal slaughter and marketing practices may increase exposure and delay detection of unusual illness [12, 14–16]

In Ghana, One Health surveillance and preparedness address on both acute threats, such as Ebola and Lassa fever, and endemic zoonoses, including rabies, anthrax, avian influenza, and zoonotic tuberculosis [17–20]. These overlapping risks highlight that effective preparedness depends not only on national policy but also on routine household practices that reduce exposure, support safe animal management, and enable timely recognition and reporting [15, 21].

The most relevant transmission interfaces often involve both wildlife exposure and household‑level livestock keeping in shared environments [15, 22]. Hunting, butchering, and environmental proximity to wildlife (e.g., fruit bats) may increase contact with zoonotic pathogens [8, 22–24]. At the same time, widespread smallholder livestock and poultry production creates frequent exposure through close cohabitation, waste handling, home slaughter, and management of sick or dead animals [14, 15]. Where household biosecurity is limited or inconsistently implemented, such routine contacts can elevate the risk of zoonotic transmission [14, 15].

Knowledge, attitudes, and practices (KAP) assessments, especially when paired with qualitative approaches, can identify misconceptions about transmission and symptom recognition, highlight social dynamics (including stigma) that undermine timely care‑seeking or reporting, and clarify which low‑cost prevention steps are most acceptable and actionable [15, 25, 26]. Despite this need, limited data exist on zoonotic disease knowledge, attitudes, and everyday livestock practices in coastal peri-urban districts of Ghana, where routine human–livestock–wildlife contact may occur [15, 17, 27].

We therefore conducted a concurrent mixed-methods study in Ada East District, Ghana, to (1) quantify community members’ knowledge, attitudes, and livestock-handling practices related to zoonotic disease risk and (2) qualitatively describe perceived transmission pathways, household biosecurity routines, and barriers to safe sick-animal management and reporting. Findings are intended to inform context-appropriate One Health education and community-level biosecurity strategies.

## Methods

### Study design and setting

We conducted a concurrent mixed-methods study integrating a quantitative cross-sectional survey with a qualitative, semi-structured focus group discussion (FGD). The study took place in Ada East District, Greater Accra Region, Ghana, in collaboration with the University of Ghana School of Public Health. Quantitative data was collected across five health facilities serving rural and peri-urban communities, including Ada East District Hospital, Kasseh Health Centre, Ada Health Centre, Pediatorkope Health Centre, and Asigbeykope Community-based Health Planning and Services (CHPS) compound. The five facilities were purposively selected to represent the major catchment areas of Ada East District and to support community‑based recruitment. Recruitment was guided by prespecified site-level enrollment targets communicated during data-collector training, and cumulative enrollment was monitored daily by the field team. Recruitment at each site continued until the site-specific target was met, and overall enrollment stopped once the planned sample size had been reached.

### Study population and eligibility

Eligible participants were community members aged ≥ 18 years who (1) currently owned and actively raised livestock (e.g., cattle, goats, pigs, sheep) and/or poultry (e.g., chickens, turkeys, ducks) at a household or smallholder scale; (2) resided within the catchment area of one of the participating health facilities; (3) could complete the survey in English or a local language used in the district (Dangme); and (4) provided written informed consent. Individuals engaged exclusively in large-scale commercial livestock production were excluded. Dangme is one of the local languages spoken in the East Ada region of Ghana. It is the dominant indigenous language of the Ada people. Research assistants were recruited based on their ability to speak both English and Dangme.

### Quantitative data collection

Before data collection, the study team met with leadership at each facility to describe the study, obtain administrative approval, and plan on‑site logistics. Trained data collectors recruited participants in outpatient waiting areas and other high-traffic areas within the facility’s catchment communities. Potential participants were identified with the assistance of local community leaders, and were approached face‑to‑face, screened for eligibility, and provided with an information sheet in English or a local language (Dangme). Interested individuals were escorted to a private space where the study was explained in detail, and written informed consent was obtained.

The survey questionnaire was developed by the study team based on a review of published KAP instruments and guidance for KAP survey design, with selected items adapted from prior Ebola/zoonoses KAP studies and additional items created to reflect the local context [15, 26, 28]. The draft instrument was pilot‑tested for clarity and feasibility (as described in the quantitative data collection procedures) and revised before field deployment. Because a validated instrument specific to this setting was not available, the questionnaire was not formally psychometrically validated.

Survey data were collected using password‑protected tablets and stored in REDCap (Research Electronic Data Capture) [29, 30]. Ten bilingual research assistants from the University of Ghana, School of Public Health, completed a two-day training that covered study procedures, informed consent, administration of the survey tool, confidentiality, and secure data handling. The questionnaire was piloted with a small group of community members drawn from non‑study facilities in the La Nkwantanang Municipal Assembly, Greater Accra Region. The pilot assessed wording, flow, and tablet functionality. Minor revisions were made to clarify question wording, standardize response options, and ensure consistency between the English and local-language versions (in language Dangme).

Interviews were interviewer‑administered in English or the participant’s preferred local language (Dangme). Data collectors read each question aloud, recorded responses directly into REDCap, and reviewed entries for completeness before submission. When internet connectivity was unavailable, responses were stored offline and synchronized to a secure REDCap server at Pennsylvania State University once connectivity was restored. No personal identifiers were stored in the analytic dataset.

### Measures

#### Sociodemographic characteristics

Participants reported age, sex, marital status, highest education level, religion, ethnicity, occupation, household size (number of people living in the household), and years of continuous residence in the community. For descriptive analyses, continuous variables were categorized based on the sample distribution to ensure meaningful group sizes and stable estimates in regression analyses. Age (20–36, 37–50, 51–85 years), household size (1–3, 4–6, ≥7 members), and years lived in the community (1–8, 9–25, 26–75 years) were grouped to enhance interpretability. Education was recorded as ≤primary, junior secondary (JS), senior secondary/technical (SS/T), or higher education. Education was dichotomized as ≤primary vs > primary. to ensure sufficient sample size within each category and stable estimates in regression analyses, while also reflecting a meaningful distinction in educational attainment in the study context. Religion was grouped as Pentecostal/Charismatic, Presbyterian, Methodist, or other; ethnicity was categorized as Ga/Dangme, Ewe, Akan, or other. Occupation was recorded in categories and grouped a priori for regression analyses into animal‑involved occupations (e.g., farming, herding, butchering, animal trading) versus non‑animal occupations/unemployed.

#### Knowledge of zoonoses

Knowledge was assessed using 25 items across four domains: (1) general zoonotic disease concepts (4 items), (2) transmission pathways (9 items), (3) EVD symptom recognition (7 items), and (4) treatment beliefs (5 items). Items used true/false/not sure response options; correct responses were scored as 1, and incorrect/not sure responses as 0. Subscale scores were calculated by summing correct responses within each domain (general 0–4; transmission 0–9; symptoms 0–7; treatment 0–5), and a total knowledge score was computed as the sum of all items (0–25). Using a modified Bloom‑type threshold (60%) [31], total scores were classified as good knowledge (≥16 of total 25) versus poor knowledge (<16 of total 25), with subscale “good vs poor” indicators defined using analogous rounded thresholds (e.g., ≥6/9 for transmission, ≥5/7 for symptoms).

#### Attitudes toward zoonoses

Attitudes were measured using eight statements that captured perceived outbreak risk and severity, vaccine acceptability, self‑efficacy in preventing infection, and anticipated stigma toward suspected cases. Response options were agree/disagree/not sure. Items were recoded into binary indicators and summed to create a composite score (0–8), with higher scores indicating more prevention‑supportive and less stigmatizing attitudes. “Not sure” responses were coded conservatively as unfavorable (0). For items reflecting fear, perceived uncontrollability, or anticipated stigma (e.g., being concerned about an outbreak, fearing death, believing outbreaks will not be contained, perceiving near‑term community outbreak risk, expecting stigmatization), disagree was coded as 1 and agree/not sure as 0. For prevention‑oriented items (e.g., willingness to accept vaccination; confidence in ability to avoid infection), agree was coded as 1 and disagree/not sure as 0. Composite scores were classified as more prevention‑supportive, less stigmatizing *(e.g.,* positive) attitudes (≥5 of total 8) versus *less* prevention‑supportive, *more* stigmatizing *(e.g.,* negative) attitudes (<5 of total 8).

#### Livestock ownership, exposures, and practices

Participants reported household ownership of animal types and whether animals were raised for income. Ownership variables were grouped into composite categories: avian livestock (any poultry species), ungulate livestock (cattle, goats, pigs, sheep), and domestic animals (rabbits, dogs, cats). Participants also reported the number of years they had raised animals for income and whether they had received formal livestock training (yes/no/not sure). Wildlife and bushmeat exposure were assessed through questions on community hunting, roadside bushmeat sales in the past year, personal bushmeat consumption in the past year, and whether fruit bats were seen near or within the home.

Practices were assessed using 13 items covering food safety, hygiene, and household-level biosecurity behaviors. Response options (‘Always’, ‘Sometimes’, ‘Never’) were scored so that protective behaviors (e.g., keeping livestock/poultry outside the home, handwashing with soap after animal contact, safe meat/surface hygiene, covering wounds, and PPE use when handling animal fluids) received 1 when reported as ‘Always’, while “Sometimes” and “Never” were scored as 0. For assessed risky behaviors (e.g., eating meat from sick animals, eating animals found dead from unknown causes, eating bushmeat or wild birds, eating fruit bitten by animals), a value of 1 was assigned when reported as ‘Never’, while “Sometimes” and “Always” were scored as 0. This conservative coding approach ensured that only consistently protective behaviors contributed positively to the composite practice score. Total practice scores ranged from 0 to 13, with scores ≥ 8 (at least 60%) classified as good practices.

### Sample size

Sample size was estimated using a single - population proportion formula [32], where by $n = {{{Z^2}p\left( {1 - p} \right)} \over {{d^2}}}$, where *Z = 1.96 * for 95% confidence, *p * is the assumed prevalence of poor KAP, and *d * is the desired margin of error. Assuming *p = 0.06* and *d = 0.03*, $n = {{{{1.96}^2} \times 0.06 \times 0.94} \over {{{0.03}^2}}} = 240.7$, which was rounded up to 241. The 6% value was specified a priori to reflect an expectation of relatively high baseline awareness, given ongoing public health messaging and routine contact with health services, and because prior local estimates were unavailable. To account for non-response and missing data, we targeted 250 participants. A total of 255 community members completed the survey.

### Quantitative analysis

Analyses were conducted in R (versions 4.2.3–4.3.1). Continuous variables were summarized using means with standard deviations (SD) or medians with interquartile ranges (IQR), and categorical variables using counts and percentages. KAP outcomes were analyzed as binary variables (good vs. poor; positive vs. negative) [26, 28].

To examine factors associated with good KAP, we fit logistic regression models with three binary outcomes: (1) good vs. poor knowledge; (2) positive vs. negative attitudes; and (3) good vs. poor livestock‑handling practices. For each outcome, we first fit univariable logistic regression models for a prespecified set of covariates, including age group, sex, education, household size, years lived in the community, occupation, and selected animal-exposure variables (e.g., livestock ownership categories, bushmeat consumption, and seeing fruit bats near the home). Multivariable logistic regression models included a priori-selected covariates based on conceptual relevance. Odds ratios (ORs) and 95% confidence intervals (CIs) were obtained by exponentiating regression coefficients. Participants with missing values on any covariate were excluded from the corresponding model using listwise deletion, resulting in an analysis based only on complete cases [33, 34].

### Spatial mapping

Participants’ community locations were geocoded and mapped to visualize the geographic distribution of survey respondents across the Ada East District. Geographic coordinates collected at interview sites were processed in ArcGIS (Esri) to map the spatial distribution of participants. To preserve anonymity, the maps display only community-level locations and do not show individual household coordinates.

### Qualitative data collection and analysis

One FGD was conducted with livestock-keeping community members (*n* = 8) who had completed the survey and indicated willingness to participate in follow-up qualitative discussions [35–37]. The FGD was held in a private room at a participating health facility and facilitated by a bilingual moderator with qualitative research experience, supported by a note-taker. The bilingual research assistants spoke English and Dangme. For the FGD, the moderator was fluent in English and Dangme, the note-taker had fluent proficiency in the two languages, and discussion among participants occurred primarily in English, with occasional use of Dangme as needed.

Before the discussion, the moderator reviewed the study purpose, ground rules, and confidentiality expectations and obtained consent for audio recording. To maintain confidentiality, participants (P) were assigned anonymized numeric identifiers (e.g., P1–P8) in transcripts and quotations.

The semi-structured discussion guide explored: (1) perceptions and understanding of zoonotic diseases (including EVD as a high‑consequence exemplar), (2) perceived pathways of animal-to-human transmission, including wildlife/bushmeat and shared environments, (3) household livestock keeping and biosecurity practices, and (4) experiences managing and reporting sick animals and preferred sources of health information. Open-ended questions were followed by targeted probes to clarify or expand upon survey findings [38]. The discussion lasted approximately 60–90 minutes, was conducted in English and Dangme as needed, and was audio-recorded and supplemented with field notes.

Audio files were transferred to a password-protected server, transcribed verbatim, and translated into English where necessary by bilingual team members [39, 40]. Transcripts were de-identified prior to analysis. We conducted thematic analysis guided by grounded theory principles [41–44]. Two researchers independently reviewed the transcript, developed an initial codebook that incorporated deductive codes from the discussion guide and inductive codes derived from the data, and then performed line-by-line coding using constant comparison. The coding team met to reconcile discrepancies and refine code definitions. Codes were organized into higher-level categories and themes, and representative quotations were selected to illustrate each theme, including both convergent and divergent viewpoints.

### Ethics approval and consent to participate

The study protocol was approved by the Ghana Health Service Ethics Review Committee (GHS-ERC: 024/08/24) and the Pennsylvania State University Institutional Review Board (STUDY00025209). The study was conducted in accordance with the ethical principles of the Declaration of Helsinki and relevant national and institutional guidelines. Administrative approval was obtained from each participating health facility. All participants provided written informed consent prior to participation. To ensure confidentiality, no direct personal identifiers were included in the analytic datasets, and all electronic files were stored on secure, password-protected servers.

## Results

### Participant characteristics

**Fig. 1:**
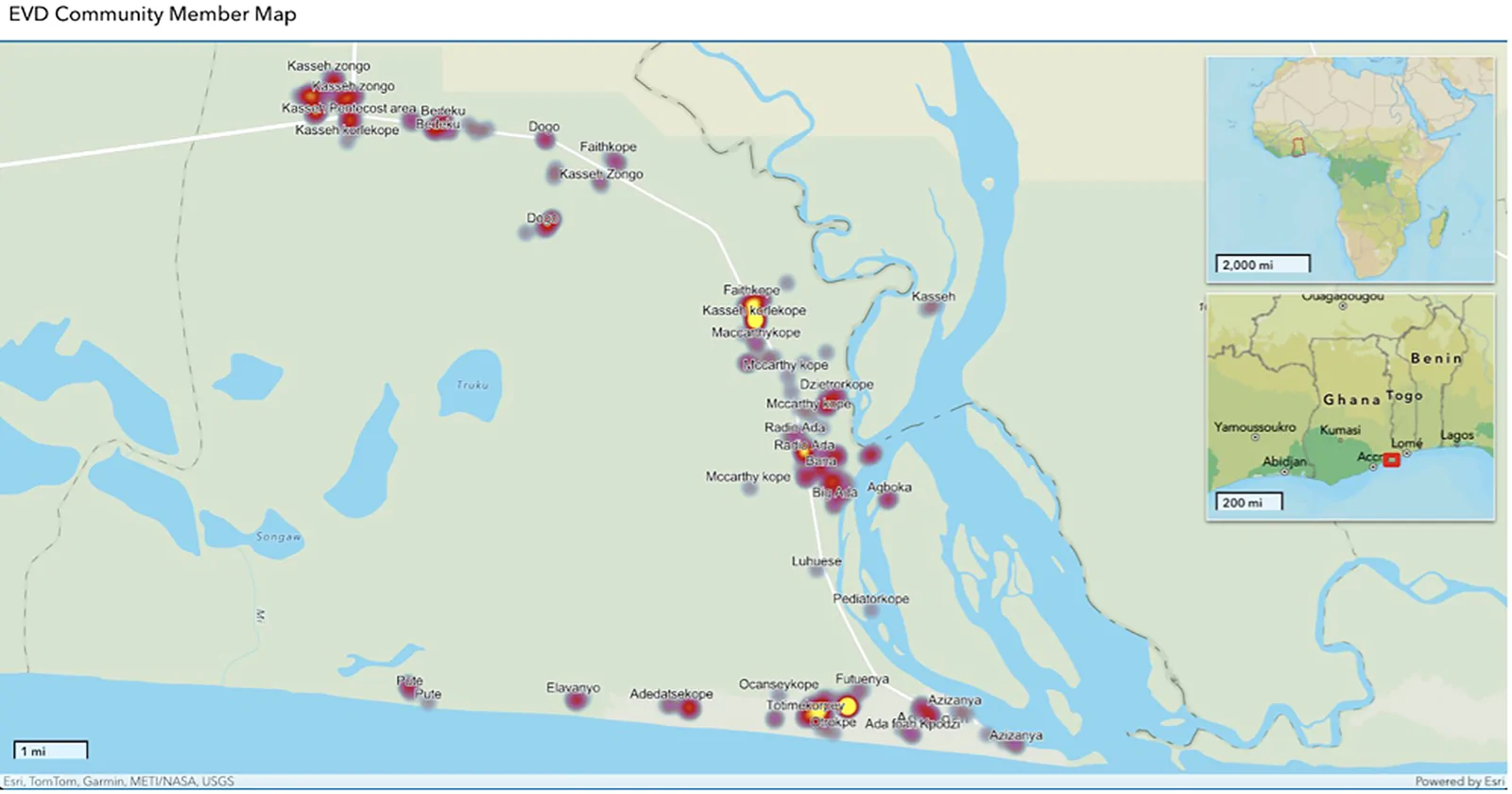
Geographic distribution of participants in Ada East District, Ghana. Caption: locations were geocoded and mapped using ArcGIS pro online v3.2 (Esri). Color gradients indicate the density of participating livestock-keeping community members, with yellow representing higher respondent density, red medium, and blue lower density. All data used are licensed for re-use in static form with attribution. Basemap and administrative boundaries source: Esri, Michael Bauer research GmbH (2023), Ghana statistical Service (ArcGIS online item f320a8af9ffa4b4baeaf1d66f03da86)

A total of 255 livestock- and/or poultry-owning community members completed the survey. Participants’ geographic distribution in the Ada East District is shown in Fig. 1. Participants, of whom 55.7% were female, had lived in their community for a mean of 19.0 years and were a mean age of 44.1 years. Households had an average of 5.9 members (SD 4.1). The predominant ethnicity was Ga/Dangme. Educational attainment was mixed, whereby 36.1% had primary education or less, 29.0% had completed junior secondary school (JS), 24.6% had senior secondary or technical education (SS/T), and 10.3% had higher education (Table 1).

**Table 1:** Sociodemographic and household characteristics of community members in Ada East District, Ghana (*N* = 255)

Sample Characteristic	N (%) N = 255
**Years Lived in Community**	19.0 (16.9)
**Years Lived in Community Group**	
0–8	94 (36.9)
9–25	82 (32.2)
26–75	79 (31.0)
**Current Age (in years)**	44.1 (13.0)
**Age Group (in years)**	
20–36	90 (35.3)
37–50	92 (36.1)
51–85	73 (28.6)
**Number of People in Household**	5.9 (4.1)
**Household Size (number of members)**	
1–3	69 (27.1)
4–6	110 (43.1)
7+	76 (29.8)
**Sex**	
Male	113 (44.3)
Female	142 (55.7)
**Education Level**	
≤Primary	91 (36.1)
Higher education	26 (10.3)
JS	73 (29.0)
SS/T	62 (24.6)
**Marital Status**	
Divorced, Widowed, or Separated	36 (14.2)
Married	157 (61.8)
Never Married, Single, or Cohabiting	61 (24.0)
**Religion**	
Methodist	23 (9.0)
Pentecostal/Charismatic	172 (67.5)
Presbyterian	29 (11.4)
Other	31 (12.2)
**Ethnicity**	
Akan	9 (3.5)
Ewe	20 (7.8)
Ga/Dangme	220 (86.3)
Other	6 (2.4)
**Occupation**	
Farmer	46 (18.1)
Herder	53 (20.9)
Laborer	23 (9.1)
Merchant	91 (35.8)
Unemployed	14 (5.5)
Other	27 (10.6)

Note: Categorical variables are n (%); continuous variables are mean (SD). Missing: education level: *n* = 3; marital status: *n* = 1; occupation: *n* = 1

### Quantitative results

#### Knowledge of zoonotic diseases

Overall, knowledge met the threshold for good knowledge in 66.3% respondents. General conceptual understanding was strong. Most participants recognized that zoonotic diseases can be transmitted from animals to humans (85.1%) and from person to person (92.9%), and that zoonotic infections can be fatal (94.1%) (Table S1).

Knowledge gaps were concentrated in specific domains. Although nearly all respondents were aware of Ebola, only 10.6% of participants met the threshold for good EVD symptom knowledge. Fewer than two-thirds correctly identified fever (58.8%) or weakness and fatigue (52.2%) as symptoms, and even fewer recognized severe headache (37.3%). Many participants incorrectly identified respiratory or non-specific complaints such as persistent cough (30.6%), sore throat (19.6%), and chills (13.3%) as characteristic of EVD (Table S1).

Regarding treatment beliefs, 85.1% indicated that zoonotic diseases can be treated with modern medicine, such as antibiotics and vaccines, and nearly nine in ten (89.4%) met the criterion for good treatment knowledge. However, a substantial minority also endorsed and believed that zoonotic diseases can be treated with herbal remedies (29.0%), traditional medicine (22.4%), home remedies (13.7%), or spiritual healing (8.2%) (Table S1).

#### Attitudes toward zoonotic diseases

Overall, 43.9% of participants were categorized as having positive attitudes toward zoonotic disease prevention and preparedness, while 56.1% had negative attitudes (Table S2). Most participants reported concern about a potential outbreak in their community (81.6%) and concern about dying from a zoonotic disease (73.7%). However, perceptions of near-term risk for their community and Ghana were mixed: 41.6% agreed that their community and Ghana were at great risk of a zoonotic disease outbreak within the next six months, while 48.6% disagreed.

Confidence in personal prevention was low, with only 63.1% agreeing that they could prevent contracting a zoonotic disease. Fewer than half (45.1%) reported that livestock or poultry deaths due to zoonotic diseases were a major personal concern, and only 20.4% indicated that inadequate zoonotic disease containment was a major concern (Table S2).

Approximately one-third (37.6%) believed that someone with a zoonotic disease would be treated differently by people in their community, and 11.8% were unsure. Despite mixed perceptions and anticipated stigma, vaccine acceptability was high: 74.9% reported willingness to accept a vaccine for a zoonotic disease such as EVD if available.

#### Livestock ownership, exposures, and practices

Most participants raised avian livestock (90.2%) and ungulate livestock (69.8%); 31.8% reported raising domestic animals, such as dogs, cats, and rabbits (Table S3). Reported direct engagement with wildlife through community hunting and roadside bushmeat sales was uncommon, although 41.6% reported seeing fruit bats near or within their home (Table S3).

Based on the composite practices score, 86.7% of participants were categorized as reporting good livestock-handling practices (Table S3). While most participants reported always washing hands after contact with dead livestock (91.4%) or animal waste (85.1%), compliance decreased for contact with live livestock (77.3%) and, in particular, after bat contact (58.4% always; 38.4% never).

Use of personal protective equipment (PPE) before contact with animal fluids was low: only 13.7% reported always using PPE, and 73.7% reported never using it. Animal housing practices indicated frequent close contact. For example, only 27.5% reported always keeping livestock and poultry outside the home, meanwhile 43.1% reported never doing so. In contrast, food safety practices were generally strong, including thorough cooking of meat (96.5% always) and cleaning raw meat preparation surfaces with soap or bleach (87.1% always) (Table S3; Additional file 1).

#### Factors associated with knowledge, attitudes, and practices

There were 66.3% who met the threshold for good zoonotic disease knowledge, 44.0% who had positive zoonotic disease attitudes, and 86.9% who reported good livestock‑handling practices (Table 2).

**Table 2:** Factors associated with good zoonotic disease knowledge (*n* = 252)

	Descriptive Analysis		Univariable Analysis			Multivariable Analysis		
Characteristic (N = 252)	Poor Knowledge *N* = 85 (34%)^1^	Good Knowledge *N* = 167 (66%)^1^	**OR**^23^	**95% CI**^3^	p-value	**aOR**^34^	**95% CI**^3^	p-value
**Age Group (years)**								
20–36	28 (31%)	62 (69%)	1.00			1.00		
37–50	27 (29%)	65 (71%)	1.09	0.58, 2.05	0.796	1.07	0.52, 2.24	0.846
51–85	30 (43%)	40 (57%)	0.60	0.31, 1.15	0.126	0.67	0.30, 1.47	0.317
**Sex**								
Male	31 (27%)	82 (73%)	1.00			1.00		
Female	54 (39%)	85 (61%)	0.60	0.35, 1.01	0.058	0.60	0.32, 1.13	0.119
**Education Level**								
≤Primary	42 (46%)	49 (54%)	1.00			1.00		
>Primary	43 (27%)	118 (73%)	**2.35**	**1.37, 4.05**	**0.002**	**1.97**	**1.05, 3.69**	**0.034**
**Occupation**								
Non-animal Job/Unemployed	50 (32%)	105 (68%)	1.00			1.00		
Animal-involved Job	35 (36%)	62 (64%)	0.84	0.50, 1.44	0.532	0.96	0.50, 1.87	0.912
**Received Formal Livestock Training**								
No	76 (34%)	147 (66%)	1.00			1.00		
Yes	9 (31%)	20 (69%)	1.15	0.51, 2.77	0.744	1.39	0.53, 4.02	0.520
**Household Size**								
1–3	27 (40%)	41 (60%)	1.00			1.00		
4–6	28 (26%)	81 (74%)	1.91	1.00, 3.66	0.051	2.04	0.98, 4.26	0.056
7+	30 (40%)	45 (60%)	0.99	0.50, 1.93	0.971	0.96	0.44, 2.09	0.926
**Years Lived in Community Group**								
1–8	33 (35%)	61 (65%)	1.00			1.00		
9–25	27 (34%)	53 (66%)	1.06	0.57, 2.00	0.851	0.90	0.42, 1.91	0.775
26–75	25 (32%)	53 (68%)	1.15	0.61, 2.18	0.673	1.31	0.61, 2.87	0.490
**Raises Avian Livestock**								
No	6 (25%)	18 (75%)	1.00			1.00		
Yes	79 (35%)	149 (65%)	0.63	0.22, 1.57	0.345	0.86	0.28, 2.36	0.773
**Raises Ungulate Livestock**								
No	28 (36%)	49 (64%)	1.00			1.00		
Yes	57 (33%)	118 (67%)	1.18	0.67, 2.07	0.558	1.19	0.63, 2.25	0.586
**Raises Domestic Animals**								
No	50 (29%)	121 (71%)	1.00			1.00		
Yes	35 (43%)	46 (57%)	**0.54**	**0.31, 0.94**	**0.029**	**0.52**	**0.28, 0.96**	**0.036**
**Eats Bushmeat**								
No	79 (32%)	165 (68%)	1.00			1.00		
Yes	6 (75%)	2 (25%)	**0.16**	**0.02, 0.71**	**0.027**	**0.06**	**0.01, 0.39**	**0.006**
**Seen Fruit bat Near or In Home**								
No	41 (28%)	105 (72%)	1.00			1.00		
Yes	44 (42%)	62 (58%)	**0.55**	**0.32, 0.93**	**0.027**	**0.53**	**0.30, 0.95**	**0.033**

Note: Values are n (row %) for participants classified as having poor vs good overall knowledge. Good knowledge was defined as a total knowledge score ≥ 16/25 (≥60% correct responses). Adjusted odds ratios (aOR) are from a multivariable logistic regression model fit using complete‑case data. CI, confidence interval

In the adjusted model for good knowledge, >primary school (vs ≤ primary) was significantly associated with higher odds of good knowledge (aOR 1.97, 95% CI 1.05–3.69). Compared with not raising domestic animals, raising domestic animals was significantly associated with a lower odds of good knowledge (aOR = 0.52, 95% CI = 0.28–0.96). Reporting bushmeat consumption in the past year was also significantly associated with lower odds of good knowledge (aOR 0.06, 95% CI 0.01–0.39), as was reporting fruit bats near or within the home (aOR 0.53, 95% CI 0.30–0.95) (Table 2)

In the adjusted model for positive attitudes, having an animal‑involved occupation (vs non‑animal job/unemployed) was significantly associated with lower odds of positive attitudes towards zoonotic diseases (aOR 0.46, 95% CI 0.25–0.85) (Table 3). A household size of 4–6 members (vs 1–3) was also significantly associated with a lower odds of positive attitudes (aOR 0.45, 95% CI 0.23–0.89). In contrast, living in the community for 26–75 years (vs 1–8 years) was associated with higher odds of positive attitudes (aOR 2.63, 95% CI 1.29–5.44).

**Table 3:** Factors associated with positive zoonotic disease attitudes (*n* = 252)

	Descriptive Statistics		Univariable Analysis			Multivariable Analysis		
Characteristic	Negative Attitudes *N* = 141 (56%)^1^	Positive Attitudes *N* = 111 (44%)^1^	**OR**^23^	**95% CI**^3^	p-value	**aOR**^34^	**95% CI**^3^	p-value
**Age Group (in years)**								
20–36	51 (57%)	39 (43%)	1.00			1.00		
37–50	54 (59%)	38 (41%)	0.92	0.51, 1.66	0.782	0.93	0.47, 1.86	0.837
51–85	36 (51%)	34 (49%)	1.24	0.66, 2.32	0.510	1.10	0.52, 2.36	0.800
**Sex**								
Man	68 (60%)	45 (40%)	1.00			1.00		
Woman	73 (53%)	66 (47%)	1.37	0.83, 2.27	0.224	1.43	0.80, 2.60	0.232
**Education Level**								
≤Primary	46 (51%)	45 (49%)	1.00			1.00		
>Primary	95 (59%)	66 (41%)	0.71	0.42, 1.19	0.195	0.68	0.36, 1.27	0.226
**Occupation**								
Non-animal Job/Unemployed	74 (48%)	81 (52%)	1.00			1.00		
Animal-involved Job	67 (69%)	30 (31%)	**0.41**	**0.24, 0.69**	**0.001**	**0.46**	**0.25, 0.85**	**0.013**
**Received Formal Livestock Training**								
No	125 (56%)	98 (44%)	1.00			1.00		
Yes	16 (55%)	13 (45%)	1.04	0.47, 2.25	0.928	1.11	0.45, 2.65	0.813
**Household Size**								
1–3	29 (43%)	39 (57%)	1.00			1.00		
4–6	71 (65%)	38 (35%)	**0.40**	**0.21, 0.74**	**0.004**	**0.45**	**0.23, 0.89**	**0.022**
7+	41 (55%)	34 (45%)	0.62	0.32, 1.19	0.152	0.65	0.31, 1.37	0.259
**Years Lived in Community Group**								
1–8 years	65 (69%)	29 (31%)	1.00			1.00		
9–25 years	44 (55%)	36 (45%)	1.83	0.99, 3.43	0.056	1.57	0.78, 3.19	0.207
26–75 years	32 (41%)	46 (59%)	**3.22**	**1.73, 6.10**	**<0.001**	**2.63**	**1.29, 5.44**	**0.008**
**Raises Avian Livestock**								
No	12 (50%)	12 (50%)	1.00			1.00		
Yes	129 (57%)	99 (43%)	0.77	0.33, 1.80	0.538	0.72	0.28, 1.84	0.485
**Raises Ungulate Livestock**								
No	40 (52%)	37 (48%)	1.00			1.00		
Yes	101 (58%)	74 (42%)	0.79	0.46, 1.36	0.396	0.88	0.48, 1.62	0.685
**Raises Domestic Animals**								
No	98 (57%)	73 (43%)	1.00			1.00		
Yes	43 (53%)	38 (47%)	1.19	0.70, 2.02	0.528	1.36	0.76, 2.46	0.300
**Eats Bushmeat**								
No	139 (57%)	105 (43%)	1.00			1.00		
Yes	2 (25%)	6 (75%)	3.97	0.89, 27.5	0.095	3.73	0.72, 28.5	0.142
**Seen Fruit bat Near or In Home**								
No	80 (55%)	66 (45%)	1.00			1.00		
Yes	61 (58%)	45 (42%)	0.89	0.54, 1.48	0.664	0.99	0.57, 1.73	0.980

Note: Values are n (row %) for participants classified as having negative vs positive attitudes. Positive attitudes were defined as a composite attitudes score ≥ 5/8, reflecting more prevention‑supportive and less stigmatizing attitudes. Crude odds ratios (OR) are from univariable logistic regression models and adjusted odds ratios (aOR) are from a multivariable logistic regression model fit using complete‑case data. CI, confidence interval; reference categories are indicated by “—”

In the adjusted model for good livestock‑handling practices, participants with animal‑involved occupations (vs non‑animal job/unemployed) had higher odds of reporting good livestock-handling practices (aOR 3.58, 95% CI 1.26–11.8) (Table 4). Bushmeat consumption was associated with substantially lower odds of good practices (aOR 0.05, 95% CI 0.01–0.34).

**Table 4:** Factors associated with good livestock handling practices (*n* = 252)

	Descriptive analysis		Univariable Analysis			Multivariable Analysis		
Characteristic	Poor Practices *N* = 33 (13%)^1^	Good Practices *N* = 219 (87%)^1^	**OR**^23^	**95% CI**^3^	p-value	**aOR**^34^	**95% CI**^3^	p-value
**Age Group (in years)**								
20–36	14 (16%)	76 (84%)	1.00			1.00		
37–50	13 (14%)	79 (86%)	1.12	0.49, 2.56	0.787	0.93	0.35, 2.49	0.880
51–85	6 (8.6%)	64 (91%)	1.96	0.74, 5.82	0.191	2.13	0.61, 8.48	0.252
**Sex**								
Male	18 (16%)	95 (84%)	1.00			1.00		
Female	15 (11%)	124 (89%)	1.57	0.75, 3.31	0.232	1.70	0.72, 4.06	0.228
**Education Level**								
≤Primary	12 (13%)	79 (87%)	1.00			1.00		
>Primary	21 (13%)	140 (87%)	1.01	0.46, 2.14	0.974	1.58	0.61, 4.13	0.343
**Occupation**								
Non-animal Job/Unemployed	25 (16%)	130 (84%)	1.00			1.00		
Animal-involved Job	8 (8.2%)	89 (92%)	2.14	0.96, 5.27	0.076	**3.58**	**1.26, 11.8**	**0.024**
**Received Formal Livestock Training**								
No	31 (14%)	192 (86%)	1.00			1.00		
Yes	2 (6.9%)	27 (93%)	2.18	0.61, 13.9	0.304	4.39	0.87, 44.1	0.125
**Household Size**								
1–3	10 (15%)	58 (85%)	1.00			1.00		
4–6	9 (8.3%)	100 (92%)	1.92	0.73, 5.09	0.183	1.62	0.54, 4.84	0.381
7+	14 (19%)	61 (81%)	0.75	0.30, 1.81	0.528	0.39	0.13, 1.12	0.087
**Years Lived in Community Group**								
1–8 years	14 (15%)	80 (85%)	1.00			1.00		
9–25 years	11 (14%)	69 (86%)	1.10	0.47, 2.63	0.830	2.22	0.79, 6.70	0.140
26–75 years	8 (10%)	70 (90%)	1.53	0.62, 4.03	0.367	3.21	1.02, 11.3	0.055
**Raises Avian Livestock**								
No	3 (13%)	21 (87%)	1.00			1.00		
Yes	30 (13%)	198 (87%)	0.94	0.21, 2.95	0.928	0.97	0.20, 3.55	0.968
**Raises Ungulate Livestock**								
No	14 (18%)	63 (82%)	1.00			1.00		
Yes	19 (11%)	156 (89%)	1.82	0.85, 3.85	0.116	1.91	0.80, 4.56	0.141
**Raises Domestic Animals**								
No	19 (11%)	152 (89%)	1.00			1.00		
Yes	14 (17%)	67 (83%)	0.60	0.28, 1.28	0.178	0.61	0.27, 1.42	0.246
**Eats Bushmeat**								
No	29 (12%)	215 (88%)	1.00			1.00		
Yes	4 (50%)	4 (50%)	**0.13**	**0.03, 0.60**	**0.006**	**0.05**	**0.01, 0.34**	**0.003**
**Seen Fruit bat Near or In Home**								
No	15 (10%)	131 (90%)	1.00			1.00		
Yes	18 (17%)	88 (83%)	0.56	0.26, 1.17	0.123	0.49	0.21, 1.12	0.094

Note: Values are n (row %) for participants classified as having poor vs good practices. Good practices were defined as a composite livestock‑handling practices score ≥ 8/13. Crude odds ratios (OR) are from univariable logistic regression models and adjusted odds ratios (aOR) are from a multivariable logistic regression model fit using complete‑case data. CI, confidence interval; reference categories are indicated by “1.00”

### Qualitative results

Eight livestock‑keeping community members participated in a focus group discussion. Thematic analysis identified three themes: (1) perceived pathways of zoonotic transmission from animals to humans, (2) household livestock keeping and biosecurity, and (3) sick animal management and reporting (Table 5).

**Table 5:** Qualitative themes and sub-themes on zoonotic risk perceptions and practices in Ada East, Ghana

Themes	Sub-themes
Perceived pathways of transmission from animals to humans	• Wildlife contact and bushmeat handling (bats, bushmeat) • Livestock contact and slaughter (including free‑ranging poultry) • Shared environments and indirect exposure (water sources; environmental beliefs including graveyards/bush burning) • Perceived high‑risk groups and settings (e.g., health workers; close cohabitation with livestock)
Household livestock keeping and biosecurity	• Routine husbandry and hygiene practices • Slaughter and food‑preparation hygiene • Risk‑reduction actions during illness (separation/isolation) • Biosecurity constraints and gaps
Sick animal management and reporting	• Initial self-management of sick animals before external help • “Watch and wait” approach to assess animal illness severity • High threshold for contacting veterinary officers • Limited early reporting of animal illness

#### Theme 1: Perceived pathways of transmission from animals to humans

Participants described multiple perceived pathways through which EVD and other zoonotic infections could spread from animals to humans. Many participants emphasized wildlife exposure—especially bats—and the handling of wild-animal meat. One participant stated,

*“I also know that Ebola virus disease spread from bats to human beings through by touching infected bats”* (P1). Another highlighted bats’ mobility as a source of concern:

*Bats can fly from here, they can fly far. So maybe it can have the disease at any other country, and it can fly to Ghana* (P3).

Bushmeat was also discussed as a potential source of infection, including concern about preparation and consumption:

*Grass cutter and then this mouse can also cause Ebola disease because they are all in the bush. Ebola disease, we heard, started from bush meat. If you bring them to the house and they are not well prepared, you are also prone to Ebola disease* (P1).

In addition to wildlife, participants discussed domestic livestock as potential sources of zoonotic infection, particularly through routine animal contact and slaughter practices. One participant described the risk from handling or slaughtering an infected animal:

*If the animal is infected and you don’t know, you go there just to feed the animal or just to slaughter some for your own purpose, and then after doing that, you are getting the sickness* (P1).

Participants sometimes linked specific animal species with heightened risk. One participant said, *“For me, it’s pig and fowl,”* (P5), elaborating that *“for pig, pork meat, I would say it’s a deadly meat. Even in our Bible and Quran, it’s been warned for us not to take pork meat. That means pork is being created to eat unwanted things like feces and so on and so forth”* (P5). The same participant raised concerns about free‑ranging poultry contacting waste: chickens can *“go to the pits to go and feed on unwanted things to come and infect us”* (P5).

Shared environments were also described as potential routes of exposure, particularly through water sources used by both animals and people. One participant explained:

*Sometimes the livestock go and drink water at the river shore. And we, the human beings, we rely on that water for cooking and so on. So, from there, the infection can pass through the animal to we, human beings* (P5).

Participants also connected water exposure to illness risk more broadly: *“because sometimes we use the river water to bath, to cook. So, from there we can attract the sickness”* (P5).

Alongside these perceived pathways, some statements reflected uncertainty or misconceptions about Ebola transmission. One participant described Ebola as airborne and emphasized washing food:

*We have to wash anything that we are going to eat because it’s an airborne disease* (P8).

Another provided broad food‑safety guidance as a preventive measure:

*We have to make sure anything we are going to eat have to be clean. If the animal is sick, we are not supposed to even slaughter and eat* (P5).

Participants also described environmental exposure beliefs, including concern about farming near graveyards: *“we don’t know what kills the ones there. So, if rain falls and erosion takes place, and we rush to the farm and bring more diseases”* (P5). This idea was further framed as a practice that should be avoided: *“we have to stop farming around the graveyards … if rain falls and erosion takes place, it brings more diseases that we can’t even mention”* (P5).

Participants also identified groups they perceived to be at higher risk of infection. Health workers were commonly mentioned due to the urgency of clinical care and the delayed recognition of Ebola:

*The health workers are in haste to take care of the person [and] you just go straight to the person and then attend to them. It is later on, where you know that this person has this sickness. By then, you are also close to the sickness or that disease* (P1).

A related concern was expressed that *“Normally, our health workers they get this disease fast”* (R1). Participants also discussed the inherent risk of close cohabitation with livestock:

*It’s a risk because you are rearing them in the house. So, whenever they have the infection, it is a risk to the human being* (P3).

#### Theme 2: Household livestock keeping and biosecurity

Household livestock keeping was described as common and purposeful, with participants describing routine husbandry activities and efforts to maintain cleanliness. One participant described prioritizing hygienic animal housing and limiting animals’ movements: *“I always try to put them at a hygienic place so that they don’t go out and come in with any disease,”* (P1), adding, *“I have to disinfect the place that I rear them, and then give them a good water, good feed”* (P1). Another participant described cleaning routines and veterinary involvement as part of animal care:

*I sweep wherever they sleep. The cattle, I have their special place for eating … And the goats too, I take good care of them by … inviting the veterinarian to treat them. So, they are always healthy* (P3).

Participants also described hygiene practices around slaughtering and food preparation. One participant noted, *“Normally, we slaughter them on the table. So, you are to wash the table with soap and water … make sure the place where you are slaughtering the animals is also clean”* (P1). Another emphasized cleaning tools and surfaces:

*You will wash the place and you disinfect [with water] the place before … And the knives that you are using, always you have to make sure they are neat. You have to clean them. That is it* (P5).

Some participants described preventive care practices aimed at keeping animals healthy, including use of medications or supplements. For example, one participant comments on their preventive care practices: *“sweeping the pen and secondly, injecting them with antibiotics and maybe vitamins and so on, for their health condition”* (P5). Personal hygiene was also noted as a risk‑reduction practice, with one community member stating, “you have to wash your hands under running water” (P7). When animals became sick, participants described attempts to prevent illness from spreading within the household herd or flock through separation. One participant stated, *“If your animals are sick, one of them is sick, you separate them. You don’t let them eat the same food,”* (P4), explaining that *“the moment the other one puts the mouth on that food, it will affect all of them. So, you have to separate them”* (P4).

However, participants also described gaps in biosecurity linked to free‑ranging animals and contact with wildlife. One participant noted that poultry may feed on dead wildlife. They specifically note that *“the fowls … go out and go and feed on maybe a dead bat and maybe that dead bat is being affected with any kind of disease”* (P6). Another participant raised concerns about food sources and advised caution, specifically referencing commercially sourced frozen poultry:

*We have to be mindful of the frozen food that we take, those chicken and things … for some time we have to at least stop eating them* (P6).

#### Theme 3: Sick animal management and reporting

Participants described multiple approaches to managing sick animals, often beginning with observation and self‑management before involving veterinary services. One participant described a *“watch and wait”* approach:

*At times, you will see that the bird is sick, but not all that serious. So, because of this, we decide not to call on the veterinary officers, just to experience them for a while. And if you see that no, still, that bird is isolating itself from the others then we call on them* (P1).

A high threshold for contacting veterinary services was described, with some participants indicating they sought professional help primarily when illness was severe or beyond their ability to manage. One participant stated, *“Sometimes, if the sickness is above you, that’s where you will call the veterinary officer,”* (P4) and described use of self‑treatment: *“we treat ourselves … We inject them … we know some of the vitamins … we have some tetracycline”* (P4). Another participant described using herbal remedies marketed for animals:

*the herbal people … sell some medicine that is good for animals and fowls … whenever they are sick … for the fowls, especially, you put [it] into their water … and the goats, you open their mouth and put [it] inside … So, this is how I treated my animals* (P3).

Despite self‑management practices, participants also described circumstances where they contacted veterinary professionals for evaluation and treatment. One participant described calling veterinary officers to assess illness:

*By calling them to come and check what is disturbing the animal … Sometimes they come and examine them and they inject them and disinfect them too* (P5).

Another participant described keeping a veterinarian’s contact information and seeking care for a sick dog: *“I have a veterinary doctor’s number … one time, it got sick … I called him to come and see what is wrong … he told me some kind of medicine that he would inject for the dog”* (P6).

Participants further emphasized the importance of timely, trustworthy communication about outbreaks and requested additional education on zoonotic transmission and prevention. One participant stated, *“I think the radio presenters or the TV presenters should be concerned … when anything happens, they need to … give us information. … if we don’t listen to[the] radio or watch TV, we would not know that there is [an] outbreak of Ebola or any other disease”* (P2). The same participant explicitly identified gaps in knowledge and requested community education:

*But I don’t know how it’s spread from animals to humans. So maybe you can lecture us more about it* (P2).

These accounts collectively illustrate that community members recognize key transmission risks yet want more detailed, locally relevant guidance on how zoonotic diseases spread and how to reduce exposure in everyday livestock-keeping activities.

The qualitative findings revealed several gaps and sub-optimal practices across key KAP domains. Misconceptions regarding zoonotic transmission were common, with some participants demonstrating limited awareness of indirect transmission pathways between animals and humans. In relation to household livestock keeping and biosecurity, practices such as close human–animal contact, limited use of protective measures, and inadequate separation between living spaces and animals were frequently reported. Sub-optimal practices in the management of sick animals were identified, including delays in seeking veterinary care, reliance on informal treatments, and underreporting to relevant authorities.

## Discussion

In this concurrent mixed-methods study of livestock-keeping community members in the Ada East District, Ghana, we found that two-thirds of respondents met the threshold for good overall knowledge of zoonotic diseases, but substantial gaps in EVD symptom recognition and variable attitudes toward risk and stigma. Reported livestock-handling practices were generally favorable; however, several high-risk behaviors remained common, including limited use of PPE when handling animal fluids, inconsistent hand hygiene after bat contact, and frequent cohabitation with animals or with animal housing in proximity to living spaces. Qualitative findings provided context for these patterns: participants commonly identified bats and bushmeat as potential sources of infection and described routine hygiene practices; however, they also reported misconceptions about transmission and a high threshold for seeking veterinary care or reporting sick animals. However, given the relatively small qualitative sample, these findings should be interpreted as exploratory and may not fully capture the diversity of experiences and practices within the broader community.

### Exposure‑tailored One Health messaging to address knowledge gaps

Although two-thirds of respondents met the threshold for good overall zoonosis knowledge, the combination of these findings and low recognition of EVD symptoms has practical implications for early detection and response. Evidence from the West Africa epidemic shows that outbreak control depends not only on general awareness but also on clear, actionable guidance that communities can implement and that supports timely care‑seeking and reporting [45, 46]. In our study, most respondents (85.1%) understood that diseases can be transmitted between animals and humans and identified several animal-to-human transmission routes, but few met the threshold for good knowledge of EVD symptoms (10.6%). This gap is important because recognition of early signs is often the first step toward prompt care seeking, isolation, and reporting. In line with previous studies, the FGD suggested that community members draw on a mix of public health messages, personal experience, and local explanatory models when reasoning about Ebola and other zoonoses, reinforcing that risk communication should be locally interpretable and tied to specific actions [46].

Comparable knowledge patterns have been documented elsewhere in West Africa. In a national survey early in the Sierra Leone epidemic, awareness of Ebola was universal, yet only about 60% could correctly cite key signs and symptoms unprompted [47]. Moreover, post‑outbreak and preparedness research has repeatedly shown that misconceptions can persist even when awareness is high, including misunderstandings about transmission, such as beliefs consistent with “airborne” spread [25, 47, 48]. Together, this literature supports messaging strategies that pair basic transmission concepts with concrete, action‑oriented guidance (e.g., which symptoms to monitor, what to do first, and where to report) delivered through trusted channels and in locally accessible formats [45, 46].

In our adjusted models, education beyond primary school was associated with significantly higher odds of good knowledge. Given that individuals with education beyond primary school had higher odds of good knowledge, sensitization, and educational interventions should be preferentially directed toward those with lower educational attainment and delivered using accessible, non-text-dependent formats such as visual materials, local languages, and demonstration-based training. Such approaches are consistent with lessons from Ebola risk communication and community engagement initiatives [45, 46]

We also found that 41.6% of respondents reported fruit bats near or within the home, and household ownership of domestic animals was common; both were associated with lower odds of good knowledge in adjusted analyses. While these associations should not be interpreted causally, they align with evidence that human–bat interactions can be frequent and diverse in Ghana and that bats in Africa are recognized reservoirs for multiple viral families with zoonotic potential [22, 49]. The FGD further highlighted that proximity to wildlife can shape perceived risk in different ways, including through misconceptions about airborne transmission or environmental exposures.

These patterns suggest that households with frequent wildlife contact may not have better access to accurate information. Practical One Health education in bat-affected communities could emphasize feasible risk-reduction steps, such as handwashing after bat contact, avoiding partially eaten fruit, and handling dead animals safely. Evidence from henipavirus research indicates that contamination of food by bats (via saliva/urine) can be epidemiologically relevant, and that physical barriers can reduce bat access to food products in real-world settings [50, 51]. Importantly, guidance should acknowledge that complete avoidance of wildlife contact is often unrealistic, particularly where bats roost near homes or where livelihoods include hunting or trade [22].

### Attitudes, stigma, and trust as determinants of reporting and prevention

Attitudes toward zoonotic diseases were mixed: many participants expressed concern about outbreaks and fear of dying from a zoonotic disease, yet fewer endorsed a high perceived likelihood of an outbreak in the near term. Only 44% met the study definition of positive attitudes, which emphasized lower anticipated stigma and greater confidence in prevention. This suggests that a majority of respondents may be less likely to engage in timely health-seeking behavior or adhere to recommended preventive practices during an outbreak. These findings matter because Ebola research consistently shows that fear, distrust, and stigma can shape whether people seek care early, cooperate with response teams, or disclose symptoms and exposures [46, 47].

Notably, vaccine acceptability was high in our sample. This is consistent with outbreak‑period evidence suggesting that many communities are willing to accept biomedical prevention when it is perceived as legitimate, accessible, and delivered through trusted systems; for example, Sierra Leone survey data documented high reported willingness to accept an approved Ebola vaccine alongside ongoing misconceptions and stigmatizing attitudes [47]. In practical terms, this means that interventions should address both skills and supplies (e.g., reducing exposure) and social dynamics (e.g., stigma reduction, trust‑building, and clarity on what happens after reporting), a pairing emphasized in community engagement lessons from the West Africa epidemic [45, 46].

We showed that animal‑involved occupations were associated with lower odds of positive attitudes but higher odds of good practices. One plausible interpretation is that individuals with daily animal contact may perceive higher personal vulnerability or anticipate social consequences during outbreaks, even while maintaining stronger routine animal‑handling practices. This pattern underscores the need for One Health programming to integrate psychosocial components (risk perception, stigma, trust) with practical exposure‑reduction training, rather than assuming that information alone will shift behavior [26, 52]. Approaches that engage occupational groups as partners, rather than solely as risk vectors, are more likely to align attitudes with existing good practices and support timely reporting and cooperation during outbreaks.

### Practice gaps and feasible household biosecurity improvements

Although 87% of respondents were classified as having good livestock‑handling practices, item‑level findings and qualitative data indicated clear opportunities for improvement, particularly around personal protective equipment use, wound protection, and bat‑related hand hygiene. Such patterns are not unusual in KAP research, where knowledge and reported practices can diverge and where structural constraints often determine what is feasible day‑to‑day [26]. Recent Ghana‑focused qualitative work on smallholder biosecurity similarly shows that adoption is shaped by capability, opportunity, and motivation—including cost, time, access to inputs, and perceived necessity—not simply awareness [52].

Low‑cost, feasible strategies in household livestock settings may therefore be most effective when they target specific high‑risk moments and minimize dependence on scarce supplies. Practical options include promoting hand hygiene immediately after handling sick or dead animals and after cleaning animal waste, encouraging the use of barriers to reduce contact with animal fluids when possible, and supporting incremental housing improvements that reduce cohabitation without undermining livelihoods. This feasibility framing is supported by Ghana‑based work documenting real‑world constraints on biosecurity in smallholder and village production systems [52, 53].

The focus group also suggested that safe management of sick animals may be constrained by access, cost, and informal care pathways. Participants described home treatment and antibiotic self‑medication, with veterinary consultation often reserved for severe or persistent illness. This aligns with evidence from Ghana indicating widespread antibiotic use among livestock and poultry farmers, frequent over‑the‑counter access without prescription, and limited veterinary involvement in administration decisions [54]. Beyond antimicrobial stewardship concerns, these informal pathways can delay recognition of unusual disease events and increase exposure risk when sick animals are handled without precautions.

### Strengthening reporting and surveillance linkages at the community level

Strengthening community‑based surveillance and animal illness reporting will likely require clear, trusted, and easy-to-use reporting pathways. During the West African Ebola epidemic, community-event-based surveillance and community engagement models demonstrated that community‑generated alerts and referrals can be implemented at scale and contribute to case detection and timely response when integrated with formal systems [46, 55]. In Ghana, recent One Health research from Greater Accra highlights that intersectoral collaboration for zoonotic surveillance may be hindered by siloed organizational structures, where human health, animal health, and environmental sectors operate independently with limited coordination, communication, and data sharing [17].

In practical terms, for the Ada East District, this supports the value of establishing simple, community‑known reporting routes (e.g., a designated contact person, hotline, or formal linkage through Community-based Health Planning and Services structures) and ensuring that reporting does not produce punitive or economically harmful outcomes that would discourage cooperation—an implementation lesson echoed across the Ebola community engagement experience [46].

#### Strengths and limitations

This mixed-methods study offers new insights into zoonotic disease awareness and livestock handling practices within a West African community, addressing a significant One Health knowledge gap. The integration of a quantitative survey and a qualitative focus group enabled triangulation of findings and provided a more comprehensive context than a single-method approach [56]. Data collection was rigorous; the survey was administered using REDCap on tablets, which reduced missing data and ensured high data quality.

However, this study has limitations. The survey was cross-sectional, so associations should not be interpreted causally [57]. The use of purposive sampling and the study’s conduct within a single district (Ada East, Ghana) may limit the generalizability of the findings to broader populations or other regions of Ghana and West Africa. Nevertheless, the study provides valuable context-specific insights into zoonotic disease knowledge, attitudes, and livestock biosecurity practices within the study setting.

Practices and attitudes were self-reported and may be influenced by social desirability bias [58]. The analytic sample for regression excluded respondents with missing covariate data, and estimates for rare exposures, such as bushmeat consumption, are subject to wide uncertainty. The survey instrument was not previously validated in this population, which may limit the reliability and comparability of the findings. Another limitation is the lack of a direct measure of household wealth. Although variables such as education, occupation, and household size were included and may partly reflect socioeconomic conditions, we have no information on a direct household wealth indicator, such as an asset-based index or livestock ownership as a proxy.

Qualitative findings were derived from a single focus group discussion (FGD) comprising eight participants, which may have limited the diversity of perspectives and the transferability of the findings. Therefore, thematic saturation may not have been fully achieved, and the findings should be interpreted as exploratory. This group size is consistent with established methodological recommendations for focus groups, which typically range from six to ten participants, and is appropriate for exploring shared experiences and perspectives rather than achieving statistical representation. During the discussion, participants expressed recurring views, and no substantially new themes emerged toward the end of the session, suggesting that thematic saturation was achieved within this group. Furthermore, the participants were relatively homogeneous in terms of background and relevance to the research topic, which may facilitate earlier saturation. An additional limitation in FGDs was that some local-language comments required minor translation during transcription, which could have resulted in the loss of subtle nuances [40].

## Conclusion

Livestock-keeping community members in Ada East District exhibited strong general awareness of zoonotic diseases and reported high adoption of several protective practices. The study identified gaps in zoonotic disease knowledge and sub-optimal biosecurity practices, including limited use of personal protective equipment and inadequate separation of animals from household living spaces. Educational attainment and occupational roles seem to significantly influence KAP outcomes. Strengthening integrated One Health education, alongside practical and resource-sensitive biosecurity support and clear animal illness reporting pathways, may improve preparedness, promote early recognition and reporting, and reduce household-level zoonotic exposure. Effective implementation of these strategies will require coordinated collaboration among public health authorities, veterinary services, local government agencies, and community leaders.

## Electronic supplementary material

Below is the link to the electronic supplementary material.

Supplementary Material 1

## Data Availability

The datasets generated and/or analyzed during the current study are available from the corresponding author on reasonable request.

## Abbreviations

CHPS: Community-based Health Planning and Services
CI: Confidence interval
EVD: Ebola virus disease
FGD: Focus group discussion
GHS-ERC: Ghana Health Service Ethics Review Committee
IRB: Institutional Review Board
KAP: Knowledge, attitudes, and practices
OR: Odds ratio
aOR: Adjusted odds ratio
PPE: Personal protective equipment
REDCap: Research Electronic Data Capture
SD: Standard deviation
