# Supplementary material for: Zoonotic disease knowledge, attitudes, and livestock biosecurity practices among community members in Ada East District, Ghana: a concurrent mixed-methods study
Source: One Health Outlook. 2026 Jun 16;8:44. doi: 10.1186/s42522-026-00221-8 (PMC13520415; doi:10.1186/s42522-026-00221-8)
Supplement: Supplementary file 1 [file 42522_2026_221_MOESM1_ESM.docx]

**Table S1.** Zoonotic Disease Knowledge Among Community Members (N = 255)

| **Zoonotic Disease Knowledge** | **Frequency**  **(n, %)** | **Mean**  **(SD)** | **95% CIs** |
| --- | --- | --- | --- |
| **Total Zoonotic Disease Knowledge** | 255 (100) | 15.9 (2.75) | 15.6 - 16.3 |
| Poor Total Knowledge (≤ 60%) (0 – 15 scores) | 86 (33.7) | 12.9 (2.28) | 12.4 - 13.4 |
| Good Total Knowledge (> 60%) (16 – 25 scores) | 169 (66.3) | 17.5 (1.30) | 17.3 - 17.7 |
| **A. General Zoonotic Disease Knowledge** | 255 (100) |  |  |
| Poor Zoonotic Disease Knowledge (≤ 60%) (0 – 2 scores) | 31 (12.2) | 1.32 (0.91) | 1.00 - 1.64 |
| Good Zoonotic Disease Knowledge (> 60%) (3 – 4 scores) | 224 (87.8) | 3.83 (0.38) | 3.78 - 3.88 |
| **B. Modes of Zoonotic Disease Transmission Knowledge** | 255 (100) |  |  |
| Poor Transmission Knowledge (≤ 60%) (0 – 5 scores) | 117 (45.9) | 4.40 (0.82) | 4.25 - 4.55 |
| Good Transmission Knowledge (> 60%) (6 – 9 scores) | 138 (54.1) | 6.59 (0.70) | 6.47 - 6.70 |
| **C. Symptoms of EVD Knowledge** | 255 (100) |  |  |
| Poor EVD Symptoms Knowledge (≤ 60%) (0 – 4 scores) | 228 (89.4) | 2.40 (1.20) | 2.23 - 2.55 |
| Good EVD Symptoms Knowledge (> 60%) (5 – 7 scores) | 27 (10.6) | 5.11 (0.32) | 4.99 - 5.23 |
| **D. Zoonotic Disease Treatment Knowledge** | 255 (100) |  |  |
| Poor Treatment Knowledge (≤ 60%) (0 – 3 scores) | 27 (10.6) | 1.37 (0.74) | 1.09 - 1.65 |
| Good Treatment Knowledge (> 60%) (4 – 5 scores) | 228 (89.4) | 4.44 (0.75) | 4.35 - 4.54 |
| **Zoonotic Disease Knowledge Statements** | **True**  **N(%)** | **False**  **N(%)** | **Not Sure**  **N(%)** |
| **General Zoonotic Disease Knowledge** |  |  |  |
| ZDs can be transmitted from animals to humans | **217 (85.1)** | 14 (5.49) | 24 (9.41) |
| ZDs can be transmitted from person to person | **237 (92.9)** | 3 (1.18) | 15 (5.88) |
| ZDs can be deadly for humans | **240 (94.1)** | 0 (0) | 15 (5.88) |
| ZDs can be deadly for animals | **205 (80.4)** | 12 (4.71) | 38 (14.9) |
| **Modes of Transmission: Animals to Humans** |  |  |  |
| ZD can be transmitted by touching an infected fruit bat | **177 (69.4)** | 78 (30.6) | 0 (0) |
| ZD can be transmitted by mosquito bite | 6 (2.35) | **249 (97.6)** | 0 (0) |
| ZD can be transmitted by dog bite | 43 (16.9) | **212 (83.1)** | 0 (0) |
| ZD can be transmitted by touching an infected nonhuman primate | **78 (30.6)** | 177 (69.4) | 0 (0) |
| ZD can be transmitted by eating raw meat | **99 (38.8)** | 156 (61.2) | 0 (0) |
| **Modes of Transmission: Humans to Humans** |  |  |  |
| ZD can be transmitted by touching an infected person’s blood or fluids | **190 (74.5)** | 65 (25.5) | 0 (0) |
| ZD can be transmitted by touching clothing/bedding contaminated with fluids | **147 (57.6)** | 108 (42.4) | 0 (0) |
| ZD can be transmitted by sexual intercourse | **73 (28.6)** | 182 (71.4) | 0 (0) |
| ZD can be transmitted by eating from the same plate | 56 (22.0) | **199 (78.0)** | 0 (0) |
| **Symptoms of EVD** |  |  |  |
| Fever is a symptom of EVD | **150 (58.8)** | 105 (41.2) | 0 (0) |
| Severe headache is a symptom of EVD | **95 (37.3)** | 160 (62.7) | 0 (0) |
| Sore throat is a symptom of EVD | **50 (19.6)** | 205 (80.4) | 0 (0) |
| Persistent cough is a symptom of EVD | 78 (30.6) | **177 (69.4)** | 0 (0) |
| Weakness and fatigue is a symptom of EVD | **133 (52.2)** | 122 (47.8) | 0 (0) |
| Red eyes is a symptom of EVD | **46 (18.0)** | 209 (82.0) | 0 (0) |
| Chills is a symptom of EVD | **34 (13.3)** | 221 (86.7) | 0 (0) |
| **Zoonotic Disease Treatment** |  |  |  |
| Can be treated with traditional medicine | 57 (22.4) | **198 (77.6)** | 0 (0) |
| Can be treated with herbal medicine/plants | 74 (29.0) | **181 (71.0)** | 0 (0) |
| Can be treated with spiritual healing | 21 (8.24) | **234 (91.8)** | 0 (0) |
| Can be treated with home remedies | 35 (13.7) | **220 (86.3)** | 0 (0) |
| Can be treated with modern medicine (e.g. antibiotics, vaccines) | **217 (85.1)** | 38 (14.9) | 0 (0) |

**Caption.** This table presents the distribution of correct responses (n, %) for each of 25 true/false knowledge items covering general zoonotic disease concepts, combined transmission routes (animal-to-human and human-to-human), Ebola Virus Disease (EVD) symptoms, and treatment modalities. Subscale scores are reported as mean ± SD with 95% confidence intervals (CIs) for General Knowledge (4 items), Transmission (9 items), Symptoms (7 items), and Treatment (5 items). The total knowledge score (sum of all subscales, range 0–25) is accompanied by its overall mean ± SD (95% CI). Participants are classified as having “Good” (≥ 16/25 correct) or “Poor” (≤ 15/25 correct) overall knowledge, with frequencies and percentages for each category. Subscale-level distributions (number and percent classified as “Good” or “Poor”) are also displayed. Knowledge items are grouped by conceptual domain, and responses are coded as “TRUE” (participant affirmed the item), “FALSE” (participant did not select the item), or “Don’t Know” (participant answered “Don’t Know”). Percentages represent the proportion of participants endorsing each statement. Of the 255 respondents, 238 had heard of Ebola, 5 were not sure, and 12 had not heard of it. Bolded indicates correct knowledge statements. Abbreviation = ZD = zoonotic disease, EVD is Ebola virus disease, ppe is personal protective equipment. Rounded to three significant figures.

**Table S2.** Community Member Attitudes Toward Zoonotic Diseases and Ebola Virus Disease (N = 255)

| **Zoonotic Disease Attitudes** | **Frequency (n, %)** | **Mean**  **(SD)** | **95% CIs** |
| --- | --- | --- | --- |
| **Total Zoonotic Disease Attitudes** | 255 (100) | 4.32 (1.53) | 4.14 - 4.51 |
| Negative Attitudes (≤ 60%) (0 – 4 scores) | 143 (56.1) | 3.20 (0.83) | 3.07 - 3.34 |
| Positive Attitudes (> 60%) (5 – 8 scores) | 112 (43.9) | 5.76 (0.89) | 5.59 - 5.92 |
| **Zoonotic Disease Attitude Statements** | **Agree**  N(%) | **Disagree**  **N(%)** | **Not Sure**  **N(%)** |
| I am concerned about a ZD outbreak in my community | 208 (81.6) | 28 (11.0) | 19 (7.45) |
| Dying from a ZD is a major concern of mine | 188 (73.7) | 67 (26.3) | 0 (0) |
| ZDs not being properly contained is a major concern of mine | 52 (20.4) | 203 (79.6) | 0 (0) |
| ZDs killing livestock and poultry is a major concern of mine | 115 (45.1) | 140 (54.9) | 0 (0) |
| I would take a vaccine for a ZD, like EVD if it existed | 177 (69.4) | 58 (22.7) | 20 (7.84) |
| My community and Ghana are at great risk for a ZD outbreak in the next 6 months | 106 (41.6) | 142 (55.7) | 7 (2.75) |
| I’m very confident that I could prevent myself from getting a ZD like EVD in the coming months | 161 (63.1) | 94 (36.9) | 0 (0) |
| I believe people in my community would treat someone who had or was suspected of having a ZD, like EVD, differently (e.g., with stigma or avoidance) | 96 (37.6) | 129 (50.6) | 30 (11.8) |

**Caption.** Summary of community attitudes regarding zoonotic disease risk, concern, stigma, prevention confidence, and vaccine willingness. Total attitude scores range from 0 to 8, with higher scores indicating more favorable attitudes (e.g., lower concern, higher confidence, lower stigma). Respondents were categorized as having “Negative Attitudes” (score ≤ 4) or “Positive Attitudes” (score > 4) based on a 60% threshold. Individual item frequencies reflect levels of agreement, disagreement, or uncertainty across eight attitude statements. Mean score, standard deviation (SD), and 95% confidence intervals (CI) are reported for total and subgroup scores.

**Table S3.** Livestock Ownership, Community-level Risks Behaviors, and Practices among Community Members (N = 255)

| **Variable** | **N = 255**^1^ | | |
| --- | --- | --- | --- |
| **Livestock Ownership** |  | | |
| **Animals Raised** |  | | |
| Avian livestock (e.g., poultry) | 230 (90.2) | | |
| Domestic animals (e.g., rabbits, dogs, cats) | 80 (31.8) | | |
| Ungulate livestock (e.g., cattle, goats, pigs, sheep) | 178 (69.8) | | |
| **Animals Raised to Earn Money** |  | | |
| Avian livestock (e.g., poultry) | 159 (62.4) | | |
| Domestic animals (e.g., rabbits, dogs, cats) | 31 (12.2) | | |
| Ungulate livestock (e.g., cattle, goats, pigs, sheep) | 171 (67.1) | | |
| **Years Raising Animals for Income** | 7.5 (8.5) | | |
| **Received Formal Livestock Training** |  | | |
| No | 224 (87.8) | | |
| Yes | 30 (11.8) | | |
| Not sure | 1 (0.4) | | |
| **Community-level Risk Behaviors** |  | | |
| **People in Community Hunts Wild Animals** |  | | |
| No | 233 (91.4) | | |
| Yes | 18 (7.1) | | |
| Not sure | 4 (1.6) | | |
| **Sean Bushmeat Sold on Roadside (in past year)** |  | | |
| No | 246 (96.5) | | |
| Yes | 8 (3.1) | | |
| Not sure | 1 (0.4) | | |
| **Seen Fruit Bat Near or In Home** |  | | |
| No | 143 (56.1) | | |
| Yes | 106 (41.6) | | |
| Not sure | 6 (2.4) | | |
|  | **Frequency**  **(n, %)** | **Mean**  **(SD)** | **95%**  **CIs** |
| **Livestock Practices** |  |  |  |
| **Total Livestock Practices** | 255 (100) | 9.35 (1.86) | 9.12 - 9.58 |
| Poor Livestock Practices (≤ 60%, 0 – 7 scores) | 34 (13.3) | 5.88 (1.39) | 5.42 - 6.35 |
| Good Livestock Practices (> 60%, 8 - 13 scores) | 221 (86.7) | 9.88 (1.26) | 9.72 - 10.05 |
| **Livestock Practice Statements** | **Good**  **(n, %)** | **Poor**  **(n, %)** | |
|  |  |  |  |
| **Protective Behaviors** | **Always** | **Sometimes** | **Never** |
| Keeps Livestock & Poultry outside of home | **70 (27.5)** | 75 (29.4) | 110 (43.1) |
| Washes hands after touching livestock | **197 (77.3)** | 45 (17.6) | 13 (5.1) |
| Washes hands after touching dead livestock | **233 (91.4)** | 13 (5.1) | 9 (3.5) |
| Washes hands after touch bats | **149 (58.4)** | 8 (3.1) | 98 (38.4) |
| Washes hands after touching animal waste | **217 (85.1)** | 26 (10.2) | 12 (4.7) |
| Wears PPE before contact with animal fluids | **35 (13.7)** | 32 (12.5) | 188 (73.7) |
| Covers wounds before animal contact | **112 (43.9)** | 59 (23.1) | 84 (32.9) |
| Washes raw meat surfaces with soap or bleach | **222 (87.1)** | 26 (10.2) | 7 (2.7) |
| Cooks meat thoroughly | **246 (96.5)** | 7 (2.7) | 2 (0.8) |
|  |  |  |  |
| **Risky Behaviors** | **Never** | **Sometimes** | **Always** |
| Eats meat from sick livestock | **220 (86.3)** | 20 (7.8) | 15 (5.9) |
| Eats meat from animals that died of unknown cause | **241 (94.5)** | 3 (1.2) | 11 (4.3) |
| Eats bushmeat or wild birds | **222 (87.1)** | 24 (9.4) | 9 (3.5) |
| Eats fruit already eaten by animals | **220 (86.3)** | 19 (7.5) | 16 (6.3) |

^1^n (%); Mean (SD)

**Caption.** This table presents (1) community patterns of livestock ownership (avian, domestic, and ungulate categories), raising livestock for income, and zoonotic‐risk behaviors observed within the community (e.g., hunting, bushmeat exposure); and (2) household‐level practices that mitigate zoonotic disease transmission (e.g., handwashing after animal contact, PPE use, safe meat handling, and avoidance of risky consumption)—each shown as n (%) or mean (SD); and (3) a stratified breakdown by “Poor Practices” versus “Good Practices,” defined as scoring ≥ 8/13 protective behaviors (≈ 60% mastery). The “Overall” column (N = 255) shows the unstratified distribution; the “Poor Practices” and “Good Practices” columns (n and %) allow us to see which ownership or exposure patterns correlate with stronger or weaker adherence to protective practices. 95% CI shown in percent. These classifications were adapted from zoonotic disease risk frameworks published by the U.S. Centers for Disease Control and Prevention (CDC) and the World Health Organization (WHO), as well as regional livestock policy guidance from the African Union Interafrican Bureau for Animal Resources (AU-IBAR), the Livestock Development Strategy for Africa (LiDeSA), and the Comprehensive Africa Agriculture Development Programme (CAADP).
